# The Treatment Results of a Standard Algorithm for Choosing the Best Entry Vessel for Intravenous Port Implantation

**DOI:** 10.1097/MD.0000000000001381

**Published:** 2015-08-21

**Authors:** Wen-Cheng Wei, Ching-Yang Wu, Ching-Feng Wu, Jui-Ying Fu, Ta-Wei Su, Sheng-Yueh Yu, Tsung-Chi Kao, Po-Jen Ko

**Affiliations:** From the Department of Surgery, Division of Thoracic and Cardiovascular Surgery (W-CW, C-YW, C-FW, T-WS, S-YY, T-CK, P-JK); and Department of Internal Medicine, Division of Chest and Critical Care, Chang Gung Memorial Hospital, Chang Gung University, Taoyuan, Taiwan (J-YF).

## Abstract

Vascular cutdown and echo guide puncture methods have its own limitations under certain conditions. There was no available algorithm for choosing entry vessel. A standard algorithm was introduced to help choose the entry vessel location according to our clinical experience and review of the literature. The goal of this study is to analyze the treatment results of the standard algorithm used to choose the entry vessel for intravenous port implantation.

During the period between March 2012 and March 2013, 507 patients who received intravenous port implantation due to advanced chemotherapy were included into this study. Choice of entry vessel was according to standard algorithm. All clinical characteristic factors were collected and complication rate and incidence were further analyzed.

Compared with our clinical experience in 2006, procedure-related complication rate declined from 1.09% to 0.4%, whereas the late complication rate decreased from 19.97% to 3.55%. No more pneumothorax, hematoma, catheter kinking, fractures, and pocket erosion were identified after using the standard algorithm. In alive oncology patients, 98% implanted port could serve a functional vascular access to fit therapeutic needs.

This standard algorithm for choosing the best entry vessel is a simple guideline that is easy to follow. The algorithm has excellent efficiency and can minimize complication rates and incidence.

## BACKGROUND

Intravenous ports are very important for patients among the oncology population. A proper intravenous port not only provides secure vascular access for all patients’ therapeutic needs, but can also reduce the frequency of venipuncture for the purposes of native vessel protection. Therefore, oncology patients’ quality of life has greatly improved since the intravenous port became available in 1982.^1^ Once the intravenous port was developed and widely available, it still required an adequate entry vessel in which to be implanted so that it could work properly. The puncture and vessel cutdown methods are the 2 most common implantation techniques for intravenous ports. A review of previous literature reveals that the vessel cutdown method is a safer procedure than the puncture method.^2,3^ The former method tries to identify the patient's own vessels, which are located in the subclavicular area. The cephalic vein is generally the target vessel, but as much as 18% of patients may not present their cephalic vein.^4^ Due to its proximity to the deltopectoral groove, the deltoid branch of the thoracoacromial vein may be used as an alternative.^5^ With the help of both of these vessels, only 5% of patients may lack an adequate native vessel in this area that could cause difficulties in intravenous port implantation.^5^ The puncture method may be required for entry vessel access in this clinical scenario.

When using the puncture method, the subclavian vein is the first to be considered as the target vessel. First, central venous access was introduced through the percutaneous puncture of the subclavian vein,^6^ which was initially used as the primary technique for intravenous port implantation. However, a variety of serious complications have been reported in previous studies regarding this method, including pinch-off syndrome,^7–9^ catheter fracture,^10–12^ pneumothorax,^8,12,13^ iatrogenic arterial puncture,^8,13,14^ hemothorax,^15^ and central vein stenosis.^16^ To prevent fatal complications, the internal jugular vein was proposed as a viable alternative due to its large caliber and superficial location. As portable echo equipment has continued to improve, surgeons became able to see the needle puncture directly through the vascular wall, thus further reducing the risk of iatrogenesis. Despite this alternative, complications have still resulted even with image guidance in the lower neck region.^17,18^ As both of these implantation methods have limitations under certain conditions, a standard algorithm was introduced to help choose the entry vessel location according to the clinical experience of the researchers and a review of the literature since 2012.^10,12,19–21^ The goal of this study is to analyze the treatment results of the standard algorithm used to choose the entry vessel for intravenous port implantation.

## MATERIALS AND METHODS

### Patients and Materials

During the period between March 2012 and March 2013, 507 patients received intravenous port implantation due to advanced chemotherapy. The survival and port functional status of the patients were followed up through July 31, 2014. B’Braun Fr.6.5 (B’Braun Medical, Chasseneuil, France) and Bard Fr.6 X port (Bard Access System Inc., UT) were the 2 types of intravenous port equipment used for implantation in this study. Catheter-related complications and instances of re-intervention were also documented. All clinical data used in this study were de-identified prior to further analysis. This study has been approved by the Ethics Committee of Chang Gung Medical Function; its institutional review board number is 100–4193A3.

### Standard Algorithm for Choosing an Entry Vessel

**FIGURE 1:**
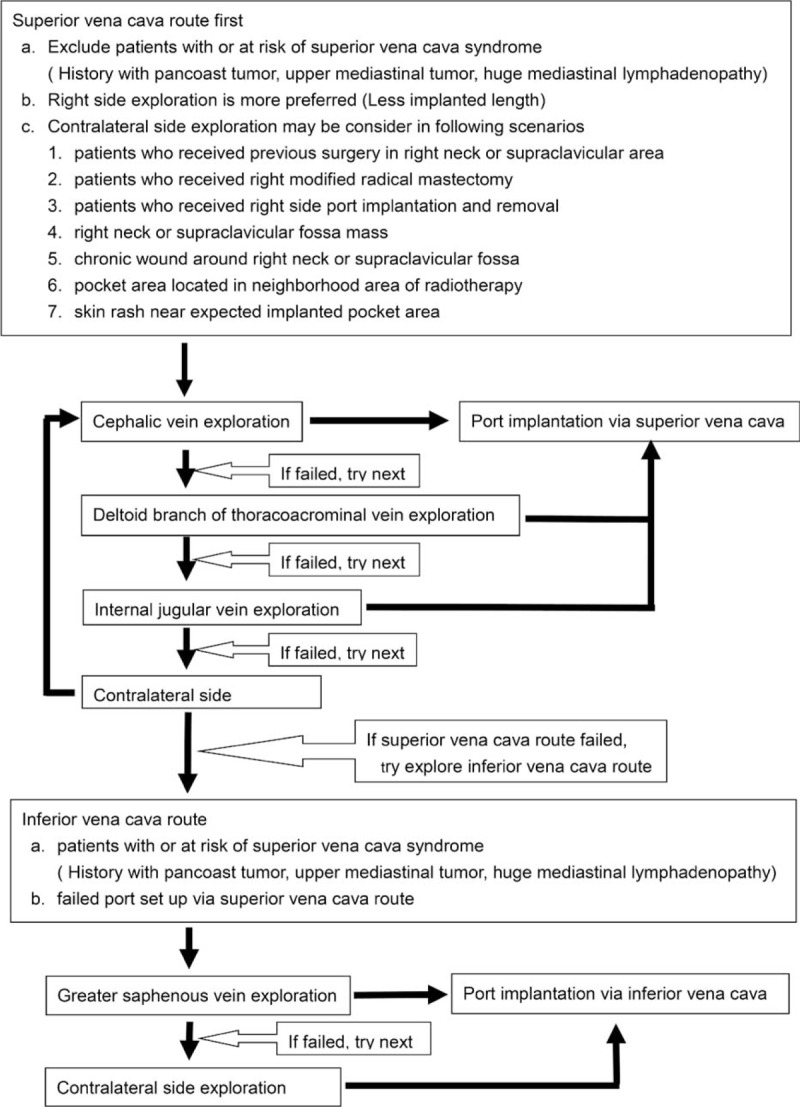
Standard algorithm for choosing the best entry vessel.

This study's standard algorithm combines the vessel cutdown and puncture implantation methods. The former is the primary approach method, in which the cephalic vein is the principal entry vessel. Patients without a cephalic vein have the alternative of using the deltoid branch of the thoracoacromial vein. Patients without both the cephalic and thoracoacromial veins had the option of the internal jugular vein for the entry choice with the use of echo guidance. The subclavian vein was not an option for catheter implantation because of the risk of iatrogenesis and the likelihood of pinch-off symptoms. Right side vessel exploration is generally preferred due to the shorter implanted catheter length and the small angulation between the axis of the catheter and the axis of the superior vena cava. Regarding patients who have had right-sided surgery in the past, such as a radical mastectomy, the left is the preferred side for vessel exploration. Furthermore, lesions located on the right side of the neck or near the supra-clavicular fossa may prevent patients from having the right internal jugular vein approach used; in such circumstances, left side exploration is preferred to eliminate the need for bilateral surgery. For patients that had or were at risk for superior vena cava syndrome, the inferior vena cava route is preferential, in which case the entry vessel was the greater saphenous vein. Either the right or left side approach was acceptable, depending on the individual surgeon's preference. The decision algorithm process is shown in Figure 1.

### Operation Method

Patients undergoing port implantation via superior vena cava route had local anesthesia performed and then a 2 cm subclavian incision made. The cephalic vein and the thoracoacromial vein's deltoid branch were explored together because both these vessels are found in the vicinity of the deltopectoral groove. Once target vessel was identified, distal ligation of the vessel and venostomy were performed. Three different catheter implantation techniques were used according to the vessel caliber and route. The vessel cutdown method is used in patients who presented with adequate vessel caliber and smooth vascular routes. The catheters were directly implanted through the venostomy site manually using fluoroscopic surveillance. Patients with a small caliber or tortuous route required wire-assisted techniques. Metallic guide wires are utilized for cannulation to establish the implantation route. The catheter may then be implanted over the wire (using the over the wire method) or with the aid of a peel-apart sheath (using the modified puncture method), with the method being determined by the diameter of the entry vessel. Patients lacking the cephalic vein and the deltoid branch of the thoracoacromial vein were approached through the internal jugular vein with echo guidance. An additional 0.5 cm small neck incision is created at the thyroid cartilage level into the underlying subcutaneous tissue prior to being punctured. Once the incision is made, the puncture needle accesses the internal jugular vein using echo image guidance from the medial aspect of the incision. After successfully puncturing the internal jugular vein, a metallic wire was inserted, a peel-apart sheath was introduced over the wire, and the catheter was implanted using fluoroscopic surveillance. An additional subcutaneous tunnel is made from the lateral aspect of the neck incision to the subclavicular incision so as to embed catheter. Once the catheter is implanted into the chosen entry vessel, it is then attached to the injection chamber. The embedded overlying subcutaneous port pocket is established between the pectoralis major fascia and the subcutaneous tissue. Having fixed the port to the pectoralis major fascia with stay suture, the wound is closed layer by layer with 3–0 dexan.

Patients undergoing port implantation via the inferior vena cava route had 2 incisions made: the subinguinal incision and then the superior inferior iliac spine incision. The purpose of the former incision is vessel exploration and that of the latter is port embedding. The greater saphenous vein is explored and the catheter is implanted using the previously described method. Once the catheter has been implanted, an additional subcutaneous tunnel is made from the subinguinal incision to the superior inferior iliac spine wound to embed the catheter. The port is fixed over the fascia of the abdominal rectus muscle. Both incisions were closed layer by layer with 3–0 dexan.

### Intraoperative Surveillance

The ideal location for the tip of the superior vena cava port was 1 cm below the carina. The acute angle between the left brachiocephalic vein and superior vena cava may require a longer incision during left side implantation to prevent the catheter from impinging on the lateral wall of the superior vena cava. The angle between the catheter axis and the superior vena axis ought to be <40 degrees.^22^ The ideal location for the tip of the inferior vena cava port is the site where the inferior vena cava and right atrium meet. This spot is easily identifiable due to the different tissue density among the internal organs. Furthermore, the movable diaphragm helps the surgeon to more precisely find the junction site. The entire procedure was carried out under fluoroscopy and the catheter tip location and port integrity were checked with plain film prior to use. A chest standing plain film and an abdomen plain film were used for the superior vena cava port and the inferior vena cava port, respectively.

### Statistics

All of the collected data were first analyzed using univariate analysis, whereas categorical variables were compared using *χ*^2^ or Fisher exact test. A *P* value <0.05 was considered statistically significant. The confidence intervals (CIs) reported are assumed to have a coverage probability of 95%. All the analyses were performed using SAS, version 9 (SAS Institute, Cary, NC).

## RESULTS

**FIGURE 2:**
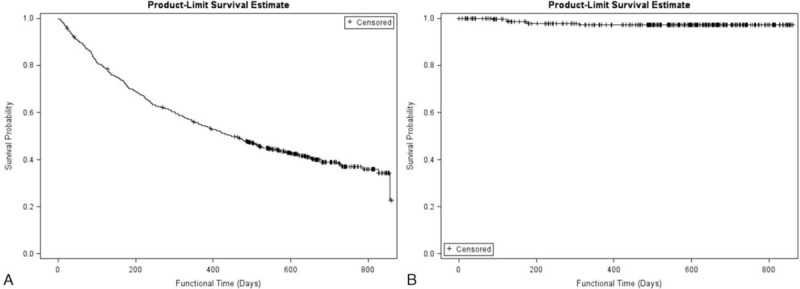
Function curves of implanted intravenous port. (A) Functional results of the implanted intravenous port in the whole population. Curve declined gradually because the patient died of cancer progress. (B) Functional results of the implanted intravenous port in patients still under surveillance. Curve declined slowly and high functional rate in alive oncology patients.

During the period of March 2012 to March 2013, 507 oncology patients underwent procedures for intravenous port implantation. In this study, more males received the implantation (317/507, 62.5%). The mean age for male and female patients was 60.9 and 57.2 years, respectively. The majority (418/507, 82.4%) of intravenous ports were implanted through the cephalic vein. In general, the operation time ranged from 36 to 52 min due to the different entry vessels used. The functional period of the ports implanted through different entry vessels varied from 289.5 to 411.6 days, but the mean functional period was 403.8 days. Table 1 shows all of the patients’ characteristics. Table 2 summarizes the reason of all reinterventions and twenty (20/ 27) of them were catheter-related complications. Catheter infection (8/20) and malfunction (6/20) were the major reason of catheter re-intervention. Only 2 (2/20) catheter migration and 3 symptomatic deep vein thrombosis (3/20) need re-operation for catheter adjustment and removal, respectively. Table 3 shows the complication rate and incidence. Compared with the functional results with previous experience in 2006, both the procedural-related and late complication rates had obviously decreased (Table 4). The procedural-related complication rate declined from 1.09% to 0.4%, whereas the late complication rate decreased from 19.97% to 3.55%. In addition, pneumothorax, hematoma, catheter kinking, fractures, and pocket erosion no longer occurred after using the standard algorithm. Furthermore, the complication rate and incidence of late complications, such as infection, malfunction, migration, and deep vein thrombosis, decreased compared with the 2006 results. Functional rate and period of an implanted intravenous port were presented in cumulative functional curve. Figure 2A revealed cumulative functional curved of all patients. The cumulative functional curve declined as patient expired due to disease progression. Figure 2B panel showed the cumulative function curve among alive oncology patients and 98% of the ports implanted were still functional and able to serve as a secure a vascular access for therapeutic needs. The difference between panel A and B revealed that cumulative functional curve of intravenous port was strongly affected by disease nature course.

**TABLE 1:**
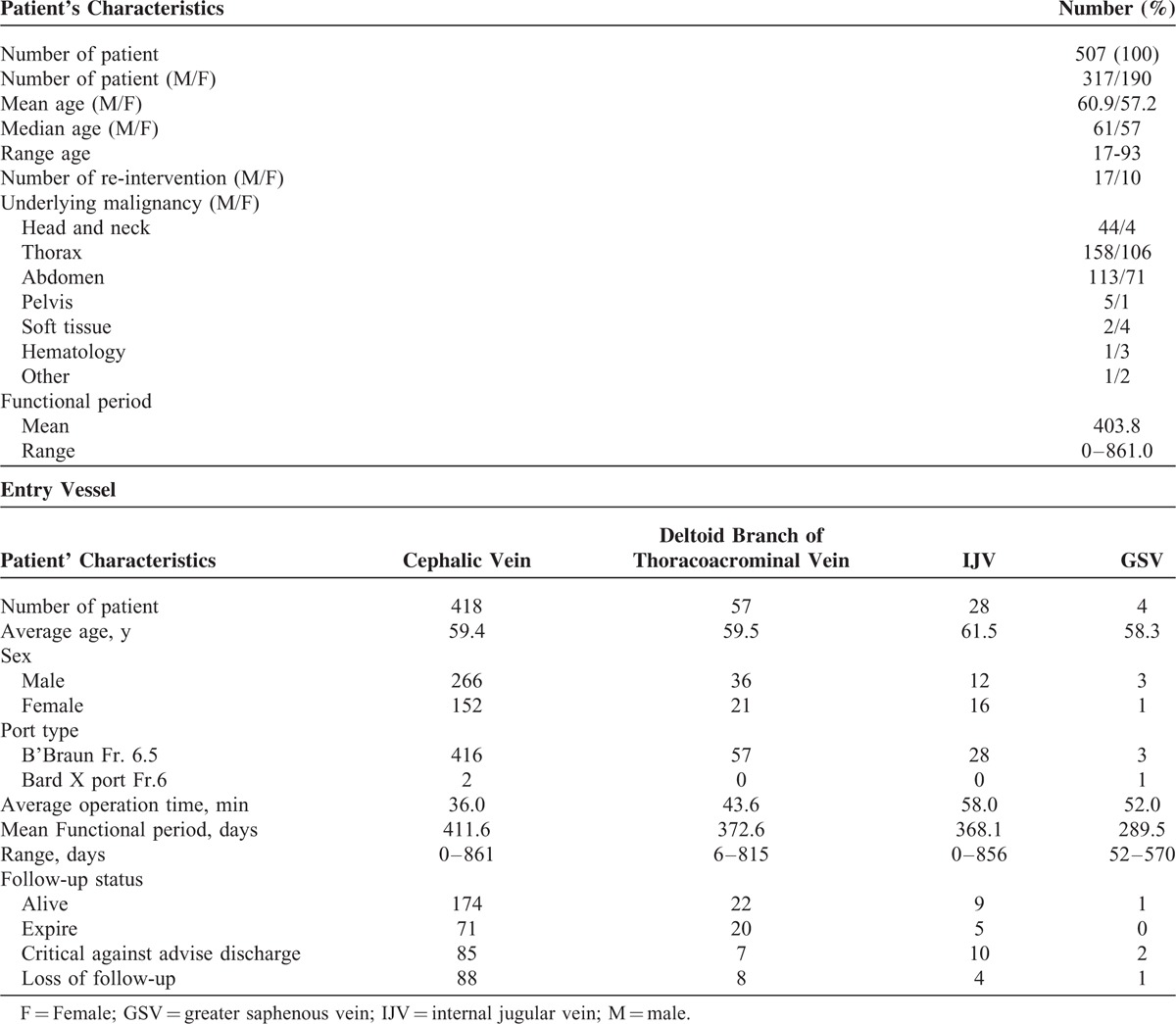
Patient Characteristics and Intravenous Port Types and Entry Sites

**TABLE 2:**
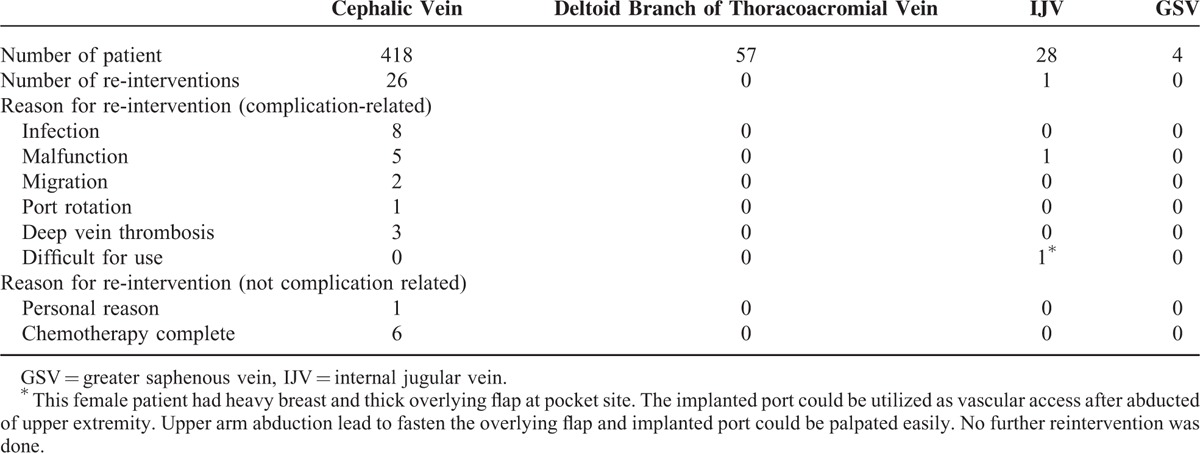
Reason for Re-intervention

**TABLE 3:**
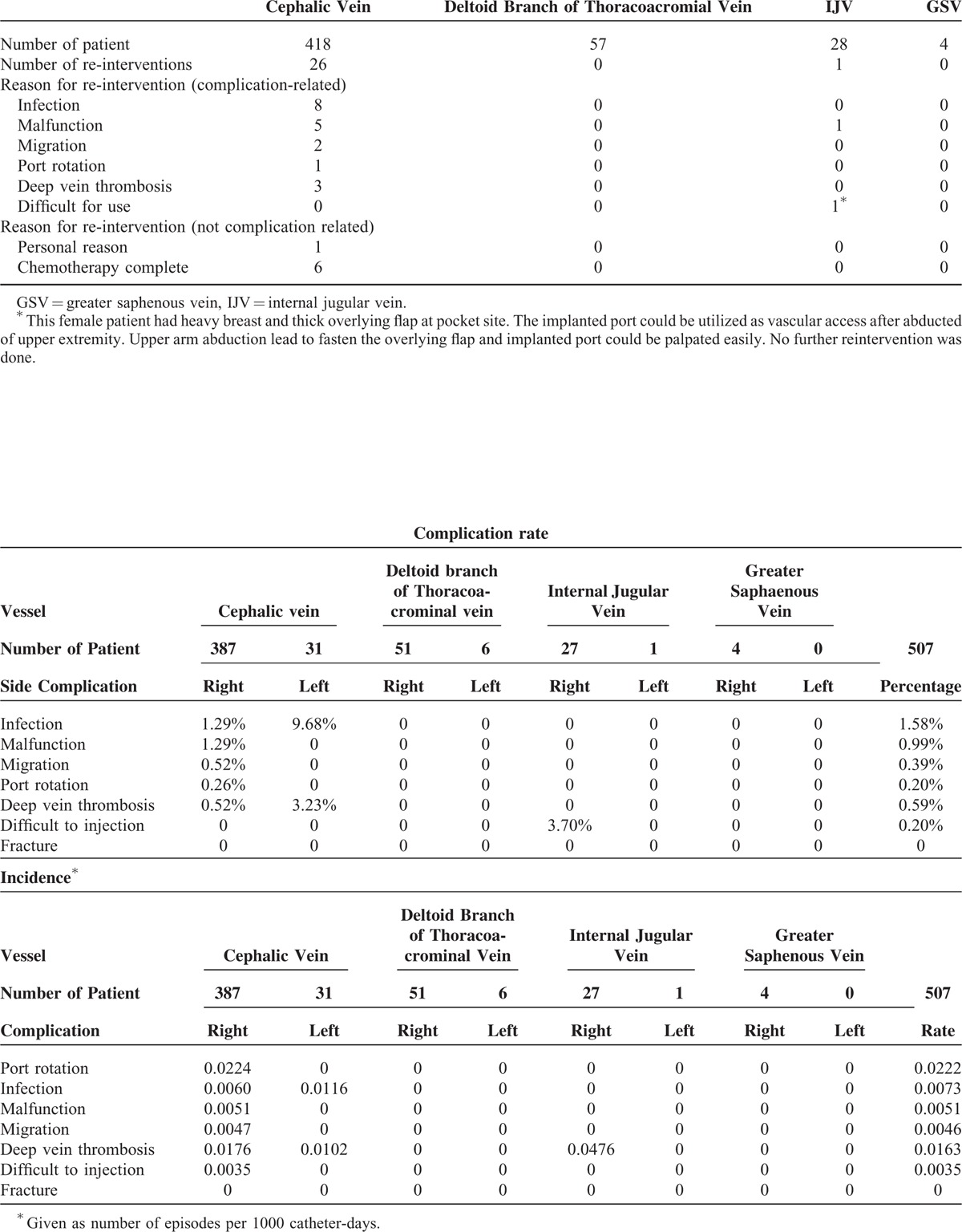
Rates and incidence of complications

**TABLE 4:**
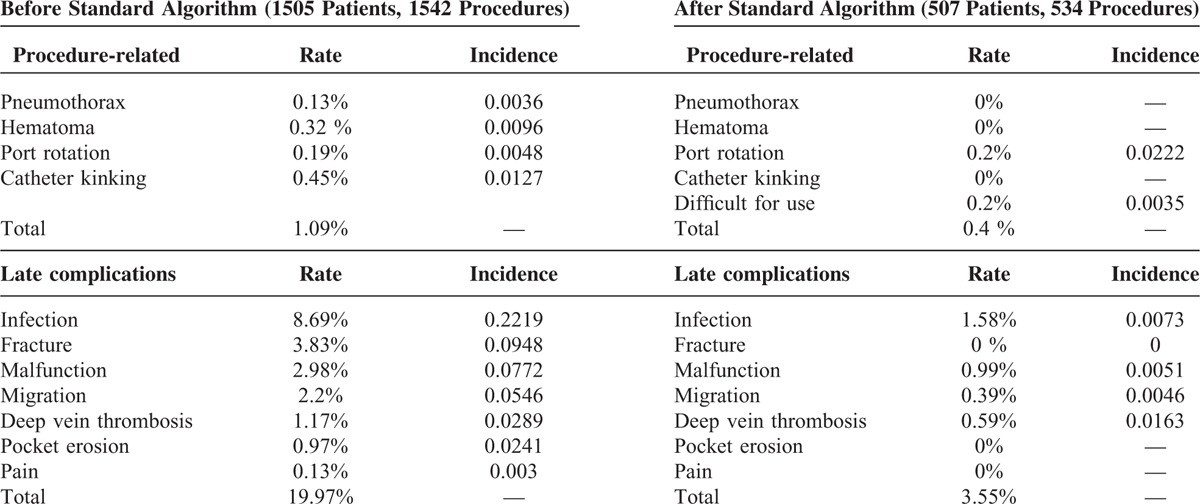
Complication Rate and Incidence Before and After Applying the Standard Algorithm for Choosing the Best Entry Vessel^12^

## DISCUSSION

Successful port implantation relies on determining the best entry vessel. Both the vessel cutdown and puncture methods can be used for catheter implantation. However, an ideal implantation method has not yet been determined and remains controversial. Di Carlo et al^3^ reviewed 45 articles and identified that the immediate complications related to percutaneous puncture and vessel cutdown were 4.5% and 0.9%, respectively. Such findings led him to recommend the vessel cutdown method for port implantations. However, in 2014, Orci et al^23^ analyzed 6 trials selected from MEDLINE, Embase, and the Cochrane Central Register of Controlled Trials and found that the safety of the percutaneous puncture method was comparable with that of the vessel cutdown method. There were no consensus for implantation method of intravenous port. Vessel cutdown method is a fast and safe technique that, when performed by experts, caused less discomfort in patients due to less tissue trauma. But catheter could not be implanted if no applicable entry vessel was identified. Puncture method could be carried out by blunt and echo guide method. However, the risk of pinch off syndrome, catheter fracture, inadvertent arterial puncture, and pneumothorax still exists if malpractice occurred. Therefore, we used the vessel cutdown method in the majority of patients and only utilized percutaneous puncture with echo guidance when a native vessel was unable to be identified. A number of vessels can be selected to be an entry route. We preferred superficial vein exploration through a single incision because it decreased tissue trauma and the risk of iatrogenic complications.^12^ Target vessels included the cephalic vein, the deltoid branch of the thoracoacromial vein, the internal jugular vein, and the greater saphenous vein, which were chosen using the previously described method. The standard algorithm for choosing the best entry vessel was developed in March 2012 and has been followed as a primary implantation principle ever since.

A review of the literature shows that the reported early complication rates ranged from 0% to 1.8% and the late complication rate ranged from 9.1% to 21.06% (Table 5 ).^2,3,8,12–14,17,18,24–33^ Furthermore, no definite recommendations had been made for patients with no cephalic vein. Several entry vessels may be considered as alternative choices, such as the basilic vein, axillary vein, external jugular vein, and femoral vein.^34–36^ However, these vessels were not appropriate due to their location. The basilica vein, axillary vein, and femoral vein are all located in deep soft tissue and thus may cause greater tissue trauma. However, the external jugular vein has a superficial location at risk for exposure due to a thin overlying flap. Therefore, a systemic decision-making algorithm is vital to ensure a high success rate, as well as to minimize complications.

**TABLE 5:**
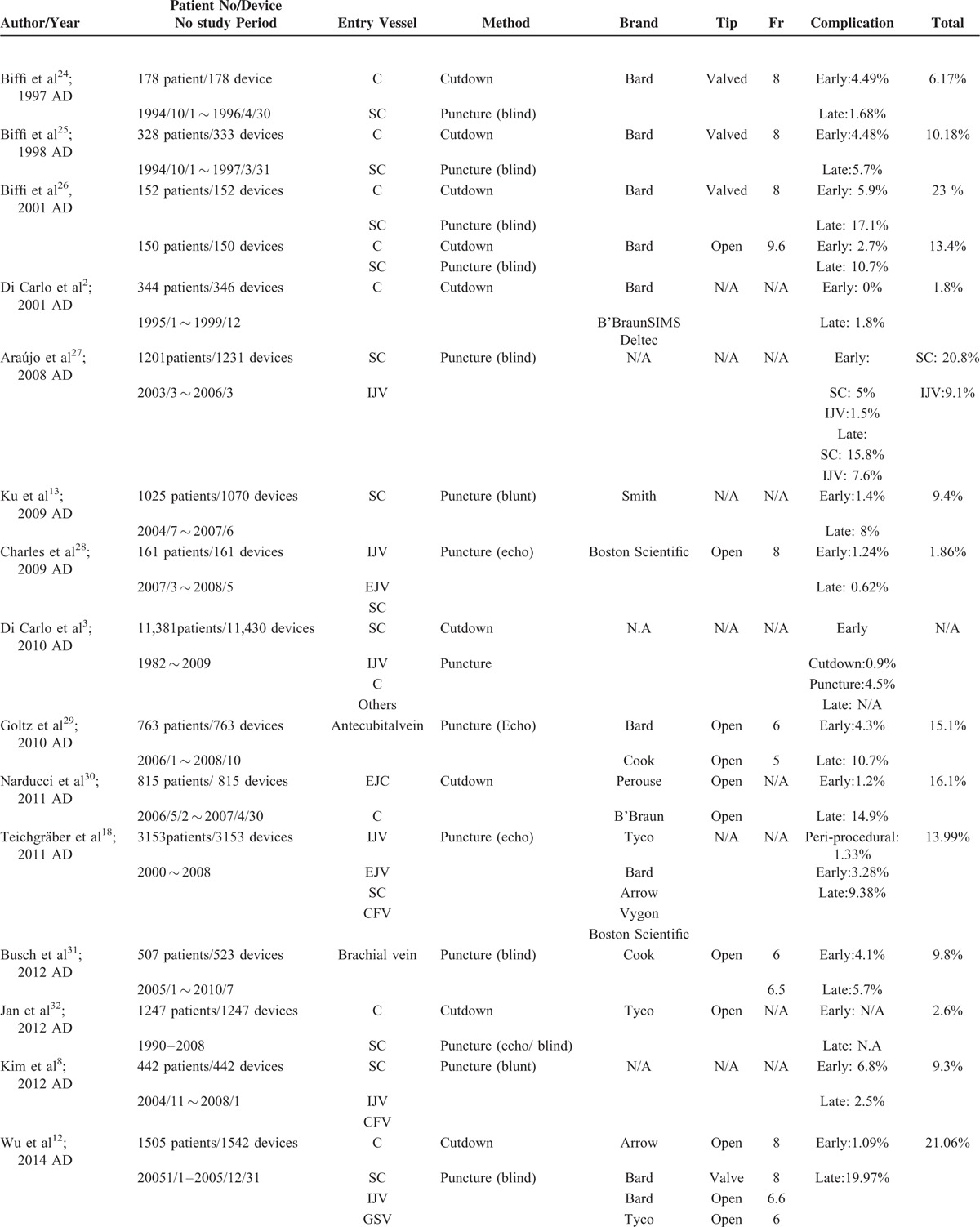
A Summary of Implantation Results From Previous Research

**TABLE 5 (Continued):**
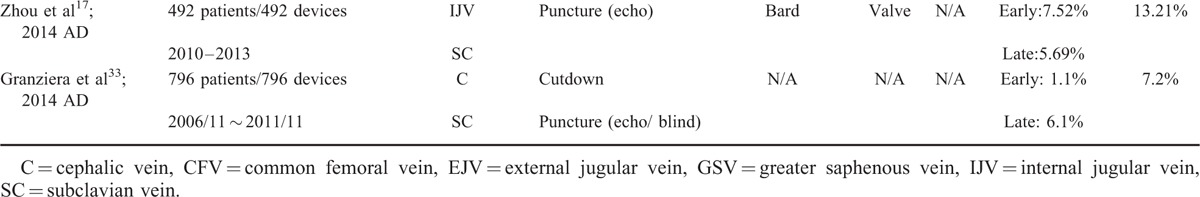
A Summary of Implantation Results From Previous Research

This study determined the standard algorithm for choosing the entry vessel (Figure 1) by summarizing the experience of our previous studies to solve 2 clinical problems, which are recommendations for choosing entry vessel according to patients’ general conditions and iatrogenic complications.^10,12,19–21^ This study found that the early and late complication rates of this study were 0.4% and 3.55%, respectively. Compared with the results of these other studies, this study demonstrates a definite algorithm that is easy to follow and offers better surgical results.

In this study, 507 patients received port implantations with the assistance of the algorithm developed herein. All patients had successful implantation with single surgery, that is, 100% successful implantation rate. In addition, low complication rates (Table 3), and a high functional rate (Figure 2) were also identified in this study. All of which evidences its clinical value for both surgeons and patients. There were no hematoma and pneumothorax identified in this study. This was due to no longer using the subclavian vein puncture, thus decreasing the risk of hematomas related to inadvertent arterial puncture, as well as the possibility of lung injury. We utilized deltoid branch of thoracoacromial vein as an alternative substitute of subclavian vein because it is located at neighborhood area of deltopectoral groove.^5^ However, varying vessel calibers and potentially tortuous routes may be encountered, which would require metallic wire to be used to establish an implantation route prior to the catheter implantation. Patients with adequate vessel caliber can have the catheter implanted over the wire. Those patients with a small vessel caliber or a tortuous route, a peel-apart needs to be used to create a subcutaneus tunnel for catheter implantation through a longitudinal split in the native vessel. Patients who had no cephalic vein and no deltoid branch of the thoracoacromial vein had the internal jugular vein chosen as the entry vessel. Implantation via internal jugular vein has 3 requirements that need to be met. The first is a puncture site high on the neck to reduce the risk of iatrogenic vessel injury and pneumothorax. This is necessary because the actual entry site is closer to the thoracic inlet even on image guidance. The lower the site puncture is, the shorter the safe distance between the entry site and the vital structure is, as well. The second point is that a small additional incision must be made at the puncture site and the underlying subcutaneous tissue must be loosened to embed the catheter. The third point involved creating a subcutaneous tunnel that goes from the lateral aspect of the neck incision to the chest to prevent catheter kinking. If these key points were followed, no complications were identified in the implantations via the internal jugular vein route. Because the subclavian vein is not used in this study, the risk of pinch-off symptoms was completely eliminated and no catheter fractures were found.

This study still has some limitations. First, this is an observational cohort study only. However, the systemic algorithm was proposed according to our previous results and then its feasibility was validated in this study. The low complication rate and incidence indicated the algorithm's efficiency. Furthermore, a large annual operation volume means that the algorithm can deal with possible vascular variations between individuals, which suggests that the algorithm could be helpful for young surgeons, as well as useful in training programs. Patients’ individual conditions are considered to choose the best entry site. Second, the vessel cutdown method made up the major components of the algorithm, followed by the utilization of a metallic wire or echo assistance that the main technique requires. As long as the anatomic structure around the entry vessel and the associated technique are familiar to the surgeon, the standard algorithm to choose the best entry vessel provides a straightforward decision for young surgeons that can further shorten operation time and decrease a patient's suffering.

## CONCLUSION

This standard algorithm for choosing the best entry vessel is a simple guideline that is easy to follow. The algorithm has excellent efficiency and can minimize complication rates and incidence.
