# The Treatment Results of a Standard Algorithm for Choosing the Best Entry Vessel for Intravenous Port Implantation: Erratum

**DOI:** 10.1097/MD.0000000000020672

**Published:** 2020-05-29

In the article, “The Treatment Results of a Standard Algorithm for Choosing the Best Entry Vessel for Intravenous Port Implantation”,^[1]^ which appears in Volume 94, Issue 33 of *Medicine*, table 3 had several incorrect cells. The correct table appears below.

**Table 3:**
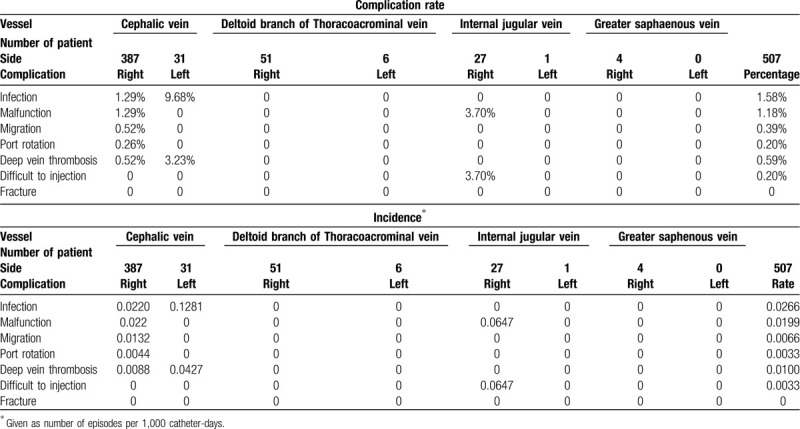
Rates and incidence of complications.

## References

[1] Wei W0C Wu C-Y Wu C-F. The Treatment Results of a Standard Algorithm for Choosing the Best Entry Vessel for Intravenous Port Implantation. *Medicine*. 2015 94:e1381. 26287429 10.1097/MD.0000000000001381 PMC4616437

